# Autobullectomy with COVID-19 in a patient with chronic obstructive pulmonary disease^☆^

**DOI:** 10.1016/j.rmcr.2023.101880

**Published:** 2023-05-31

**Authors:** Shinya Yokoe, Daisuke Kinose, Yasumitsu Ueki, Shogo Okuda, Tsukasa Nakanishi, Tomoko Iriyama, Akio Yamazaki, Satoru Kawashima, Yasuki Uchida, Hiroaki Nakagawa, Masafumi Yamaguchi, Yasutaka Nakano

**Affiliations:** Division of Respiratory Medicine, Department of Internal Medicine, Shiga University of Medical Science, Otsu, Japan

**Keywords:** Autobullectomy, Chronic obstructive pulmonary disease, Coronavirus disease 2019

## Abstract

A 72-year-old man with chronic obstructive pulmonary disease (COPD) was admitted for coronavirus disease 2019 (COVID-19). He was discharged on day 30; however, he was readmitted 6 days later due to a left lung organizing pneumonia secondary to COVID-19. After methylprednisolone treatment, the patient was discharged on day 15. One year later, computed tomography showed shrinkage of emphysematous lesions, and both total lung capacity measured using computed tomography and fraction of low attenuation volume decreased in the left lung compared to that before COVID-19. Here, we report a rare case of autobullectomy with COVID-19 in a patient with COPD.

## 1 Introduction

Emphysematous lung destruction and small airway disease often cause lung hyperinflation in patients with chronic obstructive pulmonary disease (COPD), further exacerbating dyspnea and limiting exercise capacity [1]. Surgical or bronchoscopic therapies can reduce hyperinflation and improve COPD symptoms and measurements of pulmonary function tests [2,3].

“Autobullectomy” is a rare phenomenon in which large bullas shrink, often following bacterial pneumonia, and pulmonary function eventually improves [[4], [5], [6]]. Here, we report a rare case of autobullectomy caused by coronavirus disease 2019 (COVID-19) in a patient with COPD, with analysis of lung function tests and computed tomography (CT) images pre- and post-infection.

## 2 Case presentation

**Fig. 1:**
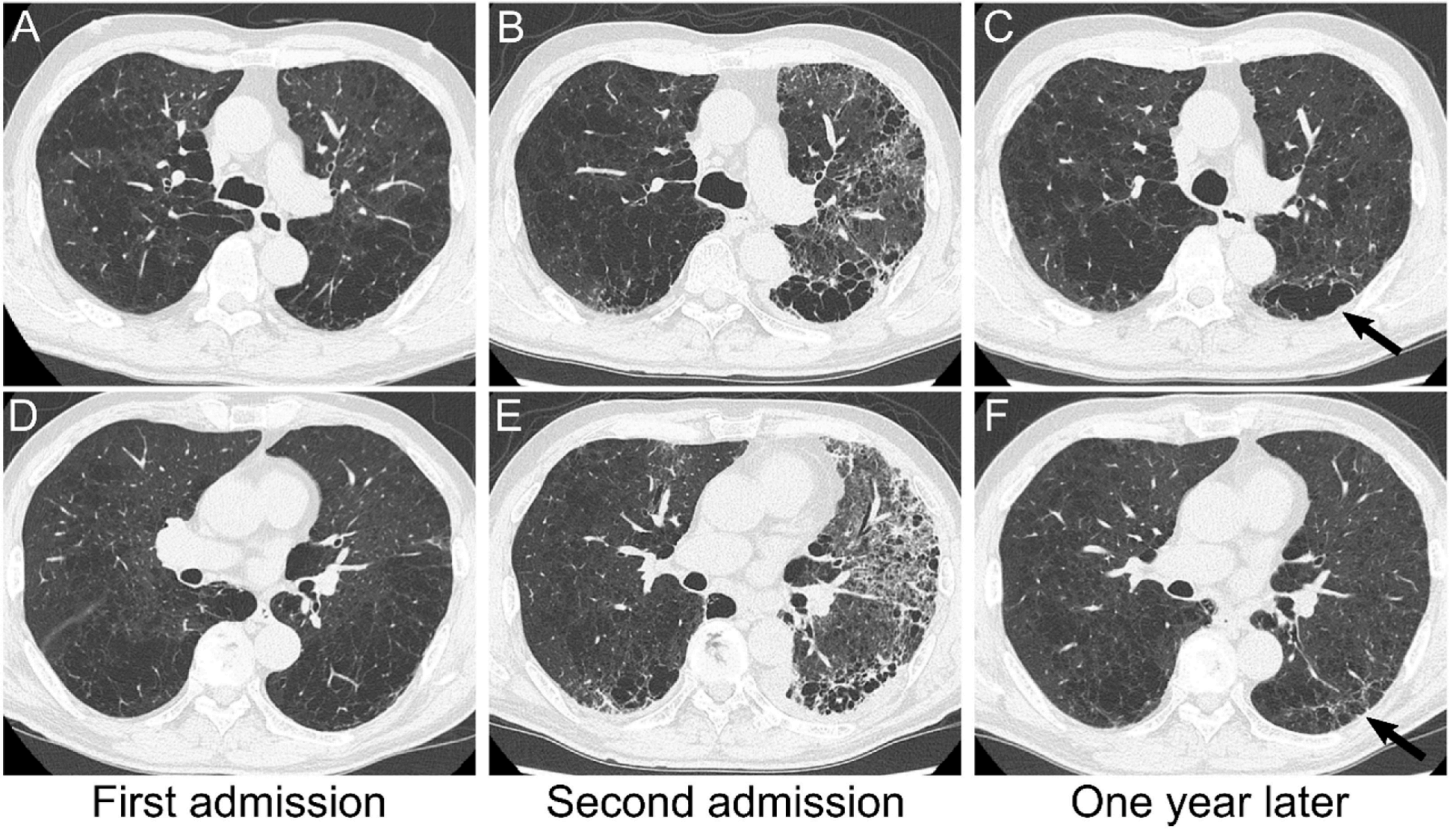
Chest CT findings on the first admission, second admission, and at one year later. (A and D) On the first admission (day 1), chest CT showed a slight increase in lung densities in the lower lobes. (B and E) On the second admission, chest CT showed opacities in the left lung. (C and F) One year after COVID-19, the emphysematous lesions in the left lung significantly shrank (arrow). CT, computed tomography; COVID-19, coronavirus disease 2019.

A 72-year-old man with COPD was admitted to our hospital due to COVID-19 in April 2021. Before COVID-19, he had a 52-pack-year smoking history with a Modified British Medical Research Council (mMRC) dyspnea grade 1 and a normal oxygenation level. He was treated with tiotropium/olodaterol. At admission, his SpO_2_ was 95% on room air. Chest CT showed a slight increase in lung density in the lower lobes in the background of severe emphysematous changes (Fig. 1 A and D). Favipiravir was administered. On day 2, his oxygenation level worsened. Oxygen (1 L/min), methylprednisolone (125 mg/day), and heparin therapies were initiated. On day 6, his oxygen level decreased further, and the chest CT images showed worsening of the condition in the lower lobes. His condition improved after tocilizumab administration, and the steroid was tapered off. He was discharged from our hospital on day 30 with home oxygen therapy (HOT).

However, he was readmitted to our hospital 6 days later because of relapsing fever. CT showed opacities in the left lung (Fig. 1 B and E). We initiated treatment with broad-spectrum antibiotics, but his oxygenation status deteriorated, and high-flow nasal cannula (HFNC) oxygen therapy (FiO_2_ 0.6) was required. β-D glucan and cytomegalovirus antigenemia test were negative. Considering organizing pneumonia caused by COVID-19 as the most likely diagnosis, we administered a high dose of methylprednisolone (1 g/day for 3 days) and tocilizumab (8mg/kg). Immediately after this intervention, his oxygen demand decreased, and chest radiograph showed improved opacity in the left lung. He was discharged 15 days after admission. Steroids were gradually tapered off after 3 months when HOT was discontinued.

Chest CT performed 1 year after COVID-19 showed shrinkage of emphysematous lesions in the left lung compared to before COVID-19 when severe organizing pneumonia due to COVID-19 had been identified (Fig. 1 C and F). In addition, pulmonary function tests after 1 year of COVID-19 showed remarkable improvement in both forced expiratory volume in 1 second (FEV_1_) and FEV_1_ (% predicted) compared to the measurements before COVID-19 (Table 1). Despite this improvement in lung function, dyspnea did not change.

**Table 1:** Lung function measurements before and after COVID-19.

Months from first admission	−19	−16	−12	−6	−5	12
FEV_1_ (L)	1.46	1.72	1.57	1.49	1.80	2.31
FEV_1_ (%predicted)	50.8	60.2	55.1	52.3	63.4	83.7

FEV_1_: forced expiratory volume in 1 s, FEV_1_ (%predicted): percentage of predicted forced expiratory volume in one second.

**Fig. 2:**
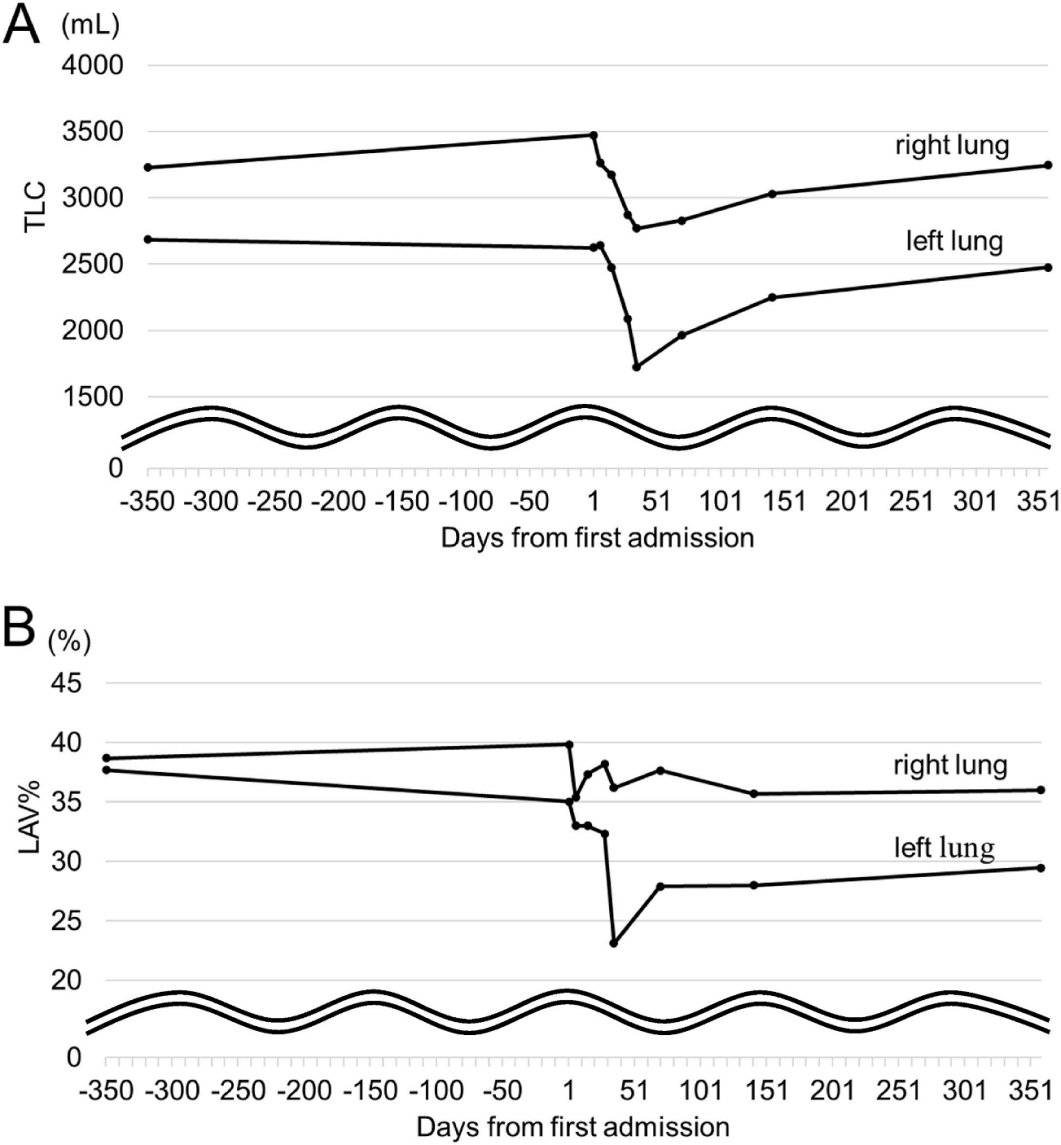
CT densitometry analysis before and after COVID-19. (A) TLC-CT decreased after COVID-19 in the left lung. (B) The fraction of low attenuation volume below −950 HU to TLC-CT (LAV%) decreased after COVID-19 in the left lung. Both reductions lasted at least a year after recovery. HU, Hounsfield unit; TLC-CT, total lung capacity measured using computed tomography.

We analyzed the CT densitometry of this patient using the VIDA LungVision software (Iowa, USA). Both total lung capacity measured using CT (TLC-CT) and the fraction of low attenuation volume below −950 HU to TLC-CT (LAV%) decreased after COVID-19 in the left lung (Fig. 2), and the reduction persisted for approximately 1 year after recovery.

## 3 Discussion

Autobullectomy occurs rarely and is primarily due to bacterial infection. After autobullectomy, shrinkage of the bulla is observed on chest radiography or CT, with improvement in pulmonary function tests [[4], [5], [6]]. Despite the pandemic, to date, there have been only a few reports of autobullectomy caused by COVID-19 [7].

Here, we present a case showing a reduction in emphysematous lesions, increased FEV_1_, and decreased TLC-CT after COVID-19. Notably, we observed a reduction of TLC-CT and LAV% after COVID-19 only on the affected side.

The pathophysiology of autobullectomy associated with bacterial pneumonia has also been inferred. When bacterial infections occur in the lungs, inflammation is evoked, leading to connective tissue deposition in the lung parenchyma [5,8]. Around emphysematous tissue, the fibrosing process may result in emphysema shrinkage [5]. It may result from inflammation in the airways, obstructing air supply to the bulla [4,6,7,9]. In our case, we observed autobullectomy that lasted for approximately 1 year in the left lung, where severe inflammation had occurred. However, this phenomenon was not observed in the right lung, which showed only slight inflammation.

Most patients with COVID-19 are asymptomatic or mildly symptomatic; however, approximately 15% develop acute respiratory distress syndrome (ARDS), resulting in lung fibrosis [10]. Although the mechanism of COVID-19-induced fibrosis is unclear, recent reports indicate that SARS-CoV-2 induces the aggregation of CD163-expressing monocyte-derived macrophages that express genes associated with profibrotic functions [11]. Moreover, there are significant similarities between COVID-19-associated macrophages and the profibrotic macrophage population of idiopathic pulmonary fibrosis [12]. Such fibrotic changes in the hyperinflated lung could shrink the emphysema and reduce air trapping, leading to decreased LAV% and TLC-CT and increased FEV_1_. We believe that these changes occurred in the present case.

TLC-CT and LAV% are important prognostic factors for COPD; therefore, interventions to reduce these can potentially improve prognosis [13,14]. Lung volume reduction surgery (LVRS) removes emphysematous lesions from the lungs and improves respiratory function, thereby improving prognosis in patients with emphysema (predominantly in the upper and rind region) and poor exercise capacity [15,16]. Bronchoscopic volume reduction (BVR) is a procedure in which a one-way valve or coil is inserted into the bronchi leading to emphysematous lesions. A large prospective randomized control trial (RCT) showed that BVR improved respiratory function and reduced dyspnea (3). Although these methods have different approaches, they all improve respiratory function and subjective symptoms by decreasing emphysematous size and hyperinflation, such as LAV% and TLC-CT.

In our case, reduced TL-CT and LAV% after COVID-19 persisted for approximately a year after recovery, which might have improved exercise capacity and reduced dyspnea. It is unclear whether autobullectomy due to COVID-19 will improve the prognosis of COPD patients because COVID-19 may result in damage to organs other than the lungs. Therefore, more case studies are needed to clarify the prognosis.

Our report is limited by the inability to fully exclude other pulmonary processes. The pneumonia at the time of readmission might have been interstitial pneumonia associated with collagen disease or drug-induced pneumonia, other than organizing pneumonia. Although we did not measure autoantibodies related to collagen diseases, we believe collagen and connective tissue diseases to be less likely because our patient had no history of collagen diseases, and no symptoms or physical findings characteristic of collagen diseases at the time of readmission, and has not developed any during follow-up since his last discharge. Moreover, he had never used drugs frequently associated with drug-induced pneumonia. On the other hand, organizing pneumonia often occurs after COVID-19. For these reasons, we consider organizing pneumonia due to COVID-19 most likely.

## 4 Conclusions

We have reported a case of autobullectomy caused by COVID-19. Autobullectomy can improve pulmonary function, however, future studies are needed to determine whether it improves prevalence and prognosis.

## Author contributions

Shinya Yokoe: Conceptualization, Methodology, Data Curation, Writing - Original Draft, Writing - Review & Editing, Visualization.

Daisuke Kinose: Conceptualization, Methodology, Data Curation, Writing - Review & Editing, Supervision.

Yasumitsu Ueki: Investigation, Data Curation.

Shogo Okuda: Investigation, Writing - Review & Editing.

Tsukasa Nakanishi: Investigation, Writing - Review & Editing.

Tomoko Iriyama: Investigation, Writing - Review & Editing.

Akio Yamazaki: Investigation, Writing - Review & Editing.

Satoru Kawashima: Investigation, Writing - Review & Editing.

Yasuki Uchida: Investigation, Writing - Review & Editing.

Hiroaki Nakagawa: Investigation, Writing - Review & Editing.

Masafumi Yamaguchi: Investigation, Writing - Review & Editing.

Yasutaka Nakano: Supervision, Writing - Review & Editing.

## Funding

This research did not receive any specific grant from funding agencies in the public, commercial, or not-for-profit sectors.

## Declaration of competing interest

The authors declare that they have no known competing financial interests or personal relationships that could have appeared to influence the work reported in this paper.
